# Accommodating persons with communication disabilities in court: Perspectives of law students

**DOI:** 10.4102/ajod.v13i0.1385

**Published:** 2024-07-08

**Authors:** Juan Bornman, Dianah Msipa

**Affiliations:** 1 Division of Speech, Language and Hearing Therapy, Department of Health and Rehabilitation Sciences, Faculty of Health, Stellenbosch University, Cape Town, South Africa; 2 Centre for Augmentative and Alternative Communication, Faculty of Humanities, University of Pretoria, Pretoria, South Africa; 3 Centre for Human Rights, Faculty of Law, University of Pretoria, Pretoria, South Africa

**Keywords:** access, accommodations, communication, court, disability, education, justice

## Abstract

**Background:**

Individuals with communication disabilities encounter obstacles in attaining equal access to justice compared to others. Despite experiencing widespread violence and abuse, they come across as challenges in seeking remedies through the legal system. One barrier is the lack of awareness among legal practitioners regarding suitable accommodations that would facilitate effective participation in court for individuals with communication disabilities.

**Objectives:**

This study explores the awareness of final-year law students concerning court accommodations available for individuals with communication disabilities, allowing them to testify in a South African court. The findings can serve as inspiration for expanding the current curriculum for law students.

**Method:**

This qualitative study used a modified six-step nominal group technique whereby participants (six law students identified through snowball sampling) generated, discussed and reached a consensus on accommodations needed by individuals with communication disabilities, enabling them to provide testimony in court. Data were analysed using thematic analysis principles.

**Results:**

The study found that although participants had not received any instruction on disability rights, access to justice or court accommodations during their legal training at the undergraduate level, they were able to perceive and learn about four main types of court accommodations for persons with communication disabilities to enable their testimony.

**Conclusion:**

Final-year law students are aware of court accommodations despite not having received formal instruction in disability law.

**Contribution:**

The inclusion of disability rights and court accommodations is recommended at the undergraduate level to ensure that when in practice, lawyers have knowledge on ensuring access to justice for persons with communication disabilities.

## Introduction

Sooner or later, most people will need a lawyer to help them navigate the legal system – be it to draft or review a contract or a will, to handle a divorce or a traffic offence, or for legal representation in court. This is even more true for persons with disabilities globally, as they typically face systemic exclusion and marginalisation that inhibit their equal participation in all major sectors of society, including the justice sector (Kim, Skinner & Parish 2020). Persons with disabilities face an elevated risk of encountering different forms of violence and abuse compared to their peers without disability (World Health Organization & World Bank 2011). A recent meta-analysis that reported on 68 studies representing 12 427 participants showed that individuals with disabilities face a notably increased risk of sexual victimisation compared to their counterparts without disabilities (odd ratio = 2.27). Moreover, sensory impairment (i.e. visual and hearing impairment) was the type of disability associated with the highest risk of sexual victimisation (Mailhot Amborski et al. 2021). This meta-analysis also showed that individuals with disabilities in African countries face significantly greater odds of sexual victimisation compared to those in all other countries.

Communication disability increases the risk of violence and abuse (Badcock & Sakellariou 2022; Larson et al. 2023; Marshall & Barrett 2018). Persons with communication disabilities have limited communication skills and cannot rely solely on speech to meet their communication needs (White et al. 2020). For some individuals, understanding language (receptive language) may be affected and for others, only the ability to express themselves verbally is affected. Persons with communication disabilities may also have various coexisting disabilities, such as intellectual, physical, sensory or multiple disabilities (Bornman 2017). Additionally, the communication disability can be present from birth or acquired later in life, for example, through a stroke. These individuals can be of any age, gender or social background. Their vulnerability to violence and abuse is heightened because communication barriers may hinder their ability to disclose and report violence and abuse (Marshall & Barrett 2018; Saxton et al. 2001).

In some cases, misinterpretation may occur when individuals lack comprehension of (sexual) assault or are unable to articulate refusal, leading to a potentially erroneous perception of consent (Benedet & Grant 2007). The intersectionality of communication disability, sexual or gender-based violence and poverty arguably make these victims some of life’s most vulnerable persons (Atewologun, 2018; De Beco 2017; Ortoleva & Lewis 2012), making it necessary for them to participate in the justice system, preferably with the assistance of a lawyer.

Crucially, research also indicates that without the right accommodations, persons with communication disabilities can struggle to participate effectively in the justice system (White & Msipa 2018). Effective communication, particularly verbal communication, is crucial in reporting human rights violations, seeking assistance and accessing legal, medical and psychosocial support (White et al. 2021). However, persons with communication disabilities may, for example, find it difficult to express themselves through speech, understand what others are saying and concentrate for long periods. Persons with communication disabilities are often denied access to fair and equal treatment in the justice system because of the numerous barriers they experience, including being discredited, stigma, being perceived as vulnerable, encountering a lack of understanding from service providers and diminished capacity to report (Marshall & Barrett 2018).

Consequently, women with communication disabilities not only have an increased risk of experiencing sexual and gender-based violence, but they also run a higher risk of receiving inappropriate responses to it (Ortoleva & Lewis 2012; World Bank 2012). A vast amount of research already exists on the nature of accommodations that may be provided to ensure that persons with communication disabilities participate effectively in the justice system. However, these accommodations can only be of practical benefit to persons with communication disabilities if the lawyers working in the justice system as prosecutors, defence counsels and magistrates are aware of them and ensure their provision. Thus, this article aims to examine how much final-year law students – who represent the next generation of lawyers – know about court accommodations for individuals with communication disabilities.

## Legal basis for the provision of accommodations to enable equal access to justice

The right to access justice is a human right enshrined in international human rights law. At a global level, Article 13 of the Convention on the Rights of Persons with Disabilities (CRPD) provides for the right to access justice, making it the first global human rights instrument to expressly provide for a substantive right to access justice (United Nations 2006). At a regional level, the right to access justice is enshrined in Article 13 of the Protocol to the African Charter on Human and Peoples’ Rights on the Rights of Persons with Disabilities in Africa (African Union 2018). South Africa has ratified both the CRPD (30 November 2007) and the African Disability Protocol (01 February 2023) indicating its willingness to uphold and be bound by the norms and standards set out in these instruments, including the right to access justice. It should be noted, however, that there is no standard definition for the term ‘access to justice’ (Nkhata 2022). Nevertheless, the meaning of access to justice can be gleaned from these instruments and from literature.

Although the CRPD and the African Disability Protocol do not define access to justice per se, they conceptualise access as a broad concept that includes social, intellectual, communicative, institutional, physical and economic accessibility while considering the diversity within disability. Access to justice, therefore, includes access to facilities and access to services, systems and procedures. The term ‘justice’ encompasses the concepts of substantive, procedural and symbolic justice. Substantive justice deals primarily with citizens and the Government. In other words, it entails an assessment of the available claims or options citizens might have if they feel that their human rights have been violated because of discrimination rooted in their disability, resulting in, for example reduced employment or education opportunities (Raj 2023). Procedural justice centres on the opportunities and barriers individuals encounter when bringing their claims to institutions of justice such as the police and the courts. Barriers include physical, structural (legal process), communicative and/or language barriers, obstacles related to information and advice, high costs, uncertainties regarding outcomes and the inadequacy of court or tribunal settings (Flynn 2016). Symbolic justice, the third component, emphasises how a specific legal system fosters a sense of belonging and empowerment among citizens. All three components of justice (namely substantive, procedural and symbolic justice) are essential and must be present to uphold the right to access justice.

However, the focus of this article is on procedural justice as this is most pertinent for effective participation by persons with communication disabilities in the justice system. Article 13(1) of the CRPD and Article 13(1) of the African Disability Protocol specifically require the provision of accommodations to ensure that persons with disabilities have equal access to justice. Indeed, the provision of accommodations is one sure way of affording procedural justice to persons with communication disabilities.

Examples of accommodations that may be provided to persons with communication disabilities can be found in the CRPD, which perceives ‘communication’ as a concept that is broader than spoken language. According to Article 2 of the CRPD, communication includes (United Nations 2006):

[*L*]anguages, display of text, Braille, tactile communication, large print, accessible multimedia as well as written, audio, plain-language, human-reader and augmentative and alternative modes, means and formats of communication, including accessible information and communication technology. (n.p.)

Therefore, if crime victims with a communication disability wish to report the incident and file charges against the alleged offender at a police station, accommodations should be put in place to ensure their effective participation. If they are denied accommodations and clear and effective communication with the police officer is not possible or if information is presented in a format that is inaccessible to the individual, then they will have been denied the right to access justice. Ultimately, they would have been discriminated against based on disability, as the denial of reasonable accommodations constitutes disability discrimination (CRPD, Article 2; African Disability Protocol, Article 1).

Moreover, because human rights are inseparable, interdependent and interconnected, the denial of access to justice also results in the denial of other human rights and fundamental freedoms (Ortoleva 2011). For example, the right to equality and non-discrimination and the right to freedom from exploitation, violence and abuse may also be violated because of a denial of the right to access justice (CRPD, Articles 5 and 16).

South Africa has not yet domesticated the CRPD and the African Disability Protocol through the enactment of disability-specific legislation despite having ratified both instruments. Nevertheless, there are provisions in South African law that can be used to protect the right of persons with communication disabilities to access justice on an equal basis with others.

The Constitution of the Republic of South Africa (1996) upholds the right to equality before the law (section 9[1]) and prohibits discrimination on various grounds including disability in section 9(3). The prohibition of disability discrimination applies to all sectors, including the justice sector, providing a basis for overcoming barriers to accessing justice through the provision of reasonable accommodations.

Moreover, the *Promotion of Equality and Prevention of Unfair Discrimination Act* (PEPUDA, 2000) prohibits discrimination based on disability, which includes denial or removal of any supporting or enabling facility (section 9a), and the failure to eliminate obstacles and to reasonably accommodate (section 9[c]). *Promotion of Equality and Prevention of Unfair Discrimination Act* can therefore also be used as a basis for providing accommodations in the justice system. However, the Constitution and PEPUDA do not state the exact provisions that can be provided to persons with communication disabilities.

Recent legal reforms are useful for bridging this gap. For example, in the past, witnesses in the South African justice system were typically expected to participate by giving oral testimony (i.e. ‘*viva voce*’) in open court (Bekker 1994). As a result of the difficulties that persons with communication disabilities have with verbal communication, their participation in legal matters was restricted. Fortunately, South African lawmakers demonstrated their commitment to correcting this violation of human rights through section 7 of the *Criminal and Related Matters (Amendment) Act 12 of 2021*, which now allows any form of communication to be considered ‘viva voce’ should the person have disabilities that render them unable to speak. Hence, this legislation closely aligns with the CRPD, which directs State Parties, including South Africa, to establish suitable mechanisms for supporting individuals in exercising their rights. This involves facilitating their effective participation in court through the provision of appropriate accommodations (White 2021; White et al. 2020; White et al. 2021).

Furthermore, the *Criminal and Related Matters (Amendment) Act* amends section 170A of the *Criminal Procedure Act* to allow witnesses with physical, psychological or mental disabilities to testify through an intermediary, not just witnesses below the age of 18 years as was the case before. Extending the use of intermediaries (communication facilitators) to persons with these disabilities may also benefit persons with communication disabilities. The *2021 Amendment Act* also provides for the use of intermediaries in proceedings other than criminal proceedings, thereby extending the accommodation of testifying with communication facilitation through an intermediary to other proceedings including civil and administrative proceedings. Cumulatively, these international and domestic instruments provide a legal basis for the provision of accommodations to persons with communication disabilities in South Africa.

## Conceptual framework for the provision of accommodations

This article relies on two conceptual frameworks for the provision of accommodations for the purposes of upholding the right of persons with communication disabilities to access justice. Firstly, the social model of disability informs the provision of accommodations by acknowledging that what prevents persons with communication disabilities from participating effectively in the justice system is not the impairment alone, but the interaction between impairment and environmental and attitudinal barriers (Msipa 2021). Therefore, to guarantee equal access to justice, it is crucial to offer accommodations to people with communication disabilities that address their specific needs (e.g. requesting frequent breaks; allowing communication systems) while also addressing environmental barriers (e.g. adjusting the lighting in court; considering the placement of communication system when providing testimony) as well as confronting attitudinal barriers (e.g. misconceptions about the ability of persons with communication disabilities to testify; use of intermediaries to rephrase questions). Secondly, the article emphasises the fact that the provision of accommodations is a right to which all persons with communication disabilities are entitled, not a privilege. South Africa, therefore, through its justice system, has a duty to uphold this right in court for all persons with communication disabilities. This position is informed by the human rights model of disability, which recognises the diversity of humanity and perceives disability as part of that human diversity. The human rights model emphasises the humanity of persons with disabilities and reiterates their entitlement to all human rights and fundamental freedoms based on that humanity. Therefore, it comes as no surprise that by employing a rights-based approach to disability, there has been a consistent global rise in awareness of disability rights and the eradication of discrimination based on disability over the last two decades. This trend was galvanised when the United Nations General Assembly adopted the CRPD and its Optional Protocol on 13 December 2006, which subsequently became the most rapidly negotiated human rights treaty to date (Watson et al. 2022).

Accommodations are the best means of guaranteeing access to justice, and all persons with disabilities have the right to receive them. Therefore, this study aimed to explore the awareness of final-year law students concerning court accommodations available for individuals with communication disabilities, allowing them to testify in a South African court. The results were used to gauge law students’ awareness of possible accommodations to inspire ideas for expanding the curriculum for undergraduate students. This knowledge could pave the way for legal practitioners to request these accommodations and ensure that the accommodations are aligned with the expectations of different members of the judicial system (e.g. lawyers, advocates, prosecutors, judges and magistrates).

## Research methods and design

### Study design

A thoroughly established, multi-step, qualitative small-group interview method, namely the nominal group technique (NGT), was employed, as it facilitates a comprehensive understanding of underexplored phenomena. In this study, the unexplored phenomena were the accommodations needed by persons with communication disability to enable their testimony in court. The NGT technique employs a systematic approach to group brainstorming, aiming to attain consensus among participants (Naudé & Bornman 2021). It is valuable for systematically generating a diverse range of perspectives, as it integrates the interactive and exploratory nature of focus groups with the depth of individual reflection. Consequently, it helps circumvent potential social pressures that may influence group-based approaches (Hugé & Mukherjee 2018). As such, it also promotes input from all members in the group while preventing one participant from dominating the discussion, as sometimes happens in focus group research (Manera et al. 2019). In addition, it is a method that efficiently gathers insights and understanding regarding crucial considerations within a short timeframe.

### Setting

The study was conducted among final-year law students at one of South Africa’s research-intensive (Tier 1) universities. The Times Higher Education World University Rankings 2022 ranked this law school as one of the best law schools in Africa and among the top law schools in the world for five consecutive years (exact ranking position withheld for confidentiality reasons). The specific law school was selected as it is considered to deliver well-trained pre-service legal practitioners who can cope with practice demands.

### Study population and sampling strategy

Participants were recruited via a non-intrusive sampling method known as snowballing (Leedy & Ormrod 2020). The snowballing started with a final-year law student approaching a lecturer after having read in the mainstream media about a court case in which the victim testified using augmentative and alternative communication. This student was informed about a large ongoing research study that focussed on court accommodations for persons with communication disabilities (White 2021). When the purpose of the research was explained, the student volunteered to participate and suggested other potential participants from their network – that is a snowballing approach. This student was asked to disseminate the information letter to their networks, and if individuals in this network indicated a willingness to participate in the study, they were asked to contact one of the researchers via WhatsApp. No pressure to participate was placed on any participant. An information letter was emailed directly to the potential participants to confirm their interest.

As per the focus of the study, only one selection criterion was set: potential participants had to be in the final year of their undergraduate legal studies at this specific university. This ensured that all potential participants would have a broad understanding of the legal system in South Africa and would not have studied any disability-specific modules at the undergraduate level. As the participants were recruited from the same network and were familiar with one another, rapport was quickly established. This resulted in honest and consistent responses being given despite the sensitive nature of the topic (Horsfall et al. 2021).

The sample included 6 final-year LLB students: 4 BA (LLB) students and 2 BCom (LLB) students. These totals are in line with general NGT researchers who state that the goal for optimal participation in an NGT should be between 4 and 7 participants (Olsen 2019). The participants included 4 males and 2 females – each of them 23 years old. The home languages of the participants included Afrikaans (4), bilingual Afrikaans and English (1) and bilingual English and isiZulu (1). All had excellent proficiency in English (in reading, writing and speaking). No participants self-disclosed that they had a disability, but four indicated that they had a relative with a disability, for example, a brother diagnosed with Asperger syndrome, a cousin with a severe intellectual and communication disability, a family member with autism (relationship not specified) or a family member with intellectual disability (relationship not specified). One participant indicated that her mother was a teacher at a school for learners with special educational needs.

### Data collection

This study formed part of a larger research study and ethical approval was obtained from the Ethics Committee in the Faculty of Humanities at the relevant university before recruitment began. Prospective participants who reached out to the researchers were invited through a WhatsApp communication group following a peer’s recommendation about the study. They were asked to indicate their availability to attend the NGT group. All participants were informed that the study’s primary aim was to identify court accommodations for persons with communication disability (regardless of their role, whether as a witness or an accused).

Although the NGT session was scheduled for 90 min, it lasted 130 min. Before the NGT group started, participants were requested to complete a consent form.

A modified six-step format was used for the nominal group. The first step involved introducing both participants and moderators, along with explaining the purpose and the sequential steps that would be undertaken to establish the context. In the second step, participants were allowed to respond to the moderator’s specific question by silently generating their own ideas. They were then instructed to independently write down their ideas without consulting or discussing them with other participants. In Step 3, participants were asked to share one thought at a time in a round-robin style of feedback, which continued until all the participants had presented all their ideas. At this stage, no discussion or debate took place. Step 4 entailed a group discussion where participants had the opportunity to ask each other to clarify their ideas and Step 5 involved the voting process and a ranking of the ideas generated. Nominal group techniques typically include these five steps (Giuliani et al. 2023). However, in this modified NGT, a sixth step was included in which the ideas that had been identified were grouped into themes by the participants. There was consensus among them that the different accommodations could not be ranked. In this section of the article, we provide a detailed stepwise description of how the NGT was applied in our study.

#### Step 1: Opening statement

After welcoming all the participants to the group, the two moderators (a legal expert and a communication disability expert) introduced the concepts of communication disability and court accommodations. We used a short video clip focussed on autism spectrum disorder – ‘Amazing things happen’ – written by Alex Arnelines and assisted by Prof Tony Attwood as the ASD advisor (https://www.youtube.com/watch?v=RbwRrVw-CRo). The video is described as having:

[*T*]he aim to raise autism awareness among non-autistic audiences to stimulate understanding and tolerance in future generations. It is intended to be viewed, discussed, and shared (for free) by anyone, but especially teachers and parents.

Thereafter, a presentation provided an overview of the study, emphasising the aim and the significance of each participant’s contribution.

#### Step 2: Generating ideas

In Step 2, participants were encouraged to contemplate and document their responses to the following question on Post-it notes of various colours: ‘What do you think could be court accommodations that could be requested for persons with communication disability, to allow their equal participation in court?’ Participants were invited to do this individually and to refrain from discussing the question within the group. The reason for maintaining silence throughout this step was to guarantee that each participant could develop and articulate their individual thoughts without being unduly influenced by comments from fellow group members (Naudé & Bornman 2021). This step took approximately 5 min.

#### Step 3: Recording ideas

Responses documented in Step 2 enabled participants to capture their ideas, and moderators then affixed their Post-it notes on the wall for the entire group to observe. Participants were subsequently prompted to individually share their written responses using a ‘round-robin’ technique, without engaging in debate, and to provide clarification regarding their comments. For instance, if they had indicated ‘support person’, they were asked to explain exactly what they meant. The fact that each participant used a unique colour of Post-it notes helped them to easily identify their own postings. Post-it notes with similar ideas were stacked together to facilitate streamlined discussions based on each concept. Some duplicates contained synonyms (e.g. ‘written statements’ and ‘statement in printed format’, while some had the exact same wording, e.g. ‘interpreter’). This also ensured that the number of potential options was reduced.

#### Step 4: Discussing ideas

**FIGURE 1:**
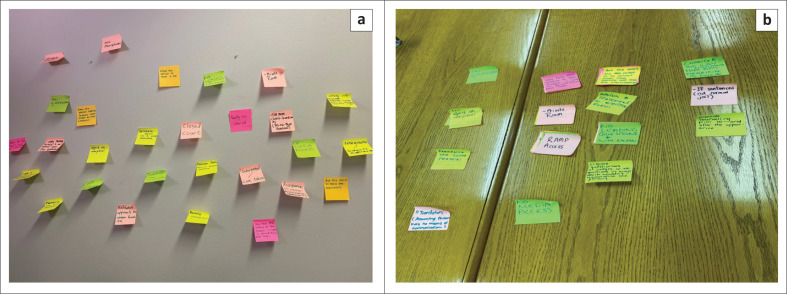
A depiction of the process to develop themes from original generated ideas. (a) Step 2: Generating ideas - participants were invited to silently generate ideas (one idea per Post-it note) and randomly place these notes on the wall. (b) Step 4: Discussing ideas - after discussing ideas, participants were asked to group ideas into main categories.

Discussing each idea documented on the Post-it notes gave participants a chance to articulate their comprehension of the rationale and the relative significance of their ideas. They subsequently engaged in discussion and debate. All participants showed high levels of interest in the topic, attentiveness to each other’s comments and engagement throughout. They were willing to provide opinions, enter into debates and disagree respectfully. As the participants were not inhibited about disagreeing with each other and were eager to explain their opinions, the nominal group discussion took longer than planned (Horsfall et al. 2021). After the discussion, participants were tasked with grouping related ideas and concepts to form the main topics. This resulted in six themes: (1) Appropriate court environment, (2) Physical accommodations, (3) Facilitating communication, (4) Questioning, (5) Remote testifying and (6) Other (see Figure 1). Each of these themes comprised several different ideas.

#### Step 5: Voting on ideas

In Step 5, participants were requested to vote to indicate whether any of the identified topics or concepts should be prioritised or removed. There was consensus among the group that all the accommodations mentioned were of importance and that none could be ranked more significant than the other. The nature of the accommodations would be determined on a case-by-case basis to ensure equality.

#### Step 6: Thematic analysis

In the final step, participants collaborated in brainstorming to categorise the generated ideas and topics into themes. The inductive approach to data analysis empowered participants to construct their own framework rooted in their individual ideas.

On concluding the NGT, the researchers adopted a latent approach that involved unpacking and describing the themes underlying the generated data (Naudé & Bornman 2021). The study concluded by thanking all participants for their lively engagement and insightful answers. They were also requested to complete a short 10-question biographic questionnaire.

### Ethical considerations

An application for full ethical approval was made to the University of Pretoria Ethics Committee in the Faculty of Humanities and ethics consent was received on 19 February 2020. The ethics approval number is GW20180718HS. Individual consent was obtained from all the participants, emphasising that their participation was voluntary and that anyone could withdraw at any time without any penalty (Mishra et al. 2018). As all participants were final-year law students, they had a full understanding of what informed consent entails. This had a positive impact on their autonomy (Barrow, Brannan & Khandhar 2023). The information letter provided details regarding the length of time needed to participate, what the study’s aims were, the procedure that would be followed and how confidentiality would be maintained. The researchers reassured the participants that their identities would be protected by providing deidentified participant numbers during the reporting of the findings. It was also reiterated that there were no risks associated with this study.

As the focus of the study is in line with the CRPD – specifically (but not limited to) Article 13 – the results may contribute to improving access to justice for persons with communication disabilities in South African courts (White et al. 2021).

### Key findings

Data obtained from the short biographic questionnaire showed that none of the participants thought that disability was addressed during their studies. However, all agreed that it should be included. One stated that it should become a core module, and another stated that after participating in the nominal group, he realised that ‘disability issues impact procedural aspects in various ways, and students should be made aware of that’. Another participant stated that ‘disability awareness is important for understanding and respecting every human being’, while another explained that ‘it is important to know how to work and accommodate individuals with disabilities to help give them access to justice’.

The data obtained through the NGT provided a wealth of information about potential accommodations that could be used in court for persons with communication disabilities. This showed that students were aware of the possible accommodations that could be offered. Initially, they grouped their ideas related to accommodations into six categories (see Table 1), namely (1) Appropriate court environment, (2) Physical accommodations, (3) Facilitating communication, (4) Questioning, (5) Remote testifying and (6) Other. The first category generated the most ideas and included the theme of ensuring an appropriate court environment. Here participants included accommodations such as holding proceedings in closed court and prohibiting media access to the courtroom. The former accommodation was featured five times, while the latter was featured only once. They also discussed aspects such as limiting the number of people present in court to only the parties who are directly involved and limiting spectator access. Furthermore, they suggested holding court in a private or separate room in the court or that the person could testify outside of court – such as the accommodation that exists for children.

**TABLE 1:** Key accommodations as identified, categorised and ranked by participants after removal of duplicate ideas (*N* = 6).

Themes	Verbatim ideas as displayed on Post-it notes
Appropriate court environment	Only have the parties to the matter in court, such as the plaintiff, defendant and interpreter Closed court Limited audience or no audience Limit the amount of people in court Ask the court to limit the spectators No media access Private room Separate room in court building Have the person testify outside court (such as with minors)
Physical accommodations	Ramp access Dimming the lights Early morning hearings – use natural lights (fewer light bulbs) No microphones Relaxed approach to certain formalities Allow breaks on a regular basis when overwhelmed Accommodate for any possible disability, e.g. Braille signs, non-fluorescent lights, etcetera. Allow for more frequent recess periods at the request of the autistic person Allow the person to wear a hat
Facilitating communication	‘Communication translators’ – assuming person has no means of communication An intermediary Acquire an interpreter (e.g. language interpreters) Interpreters (e.g. for sign language) Presence of a close person Have a guardian or caregiver accompany the person Allow family member or friend to assist if needed (help them feel calm)
Questioning	No leading questions (with exception) Use more closed questions (yes/no type questions) Easy-to-read materials Assistance in cross-examination (protection) Convey information through different modes? Such as speakers, electronic signs, etcetera. Ask the court or the judge to be lenient when they testify, and they are overwhelmed Giving a statement in written form Prepare questions or judges to ask questions in ways which will not traumatise the person
Remote testifying	Remote testifying The court should find an appropriate method of allowing the testimony (ways in which they are comfortable) Taking testimony from a person at home Testifying via video call
Other	Change the way (conditions) that bail hearing is done If sentenced, not normal jail Provide counselling prior to and after court appearance

The second theme concerned accommodations aimed at making the physical environment in the courtroom more sympathetic, for example by reducing the level of formalities, providing ramp access to the courtroom, allowing frequent breaks and using natural light instead of flickering fluorescent lights. Accommodations relating to the reduction of the amount of light for persons with autism featured the most in this category. Participants repeatedly mentioned that accommodations should not be treated as a ‘one-size-fits-all’ approach and that the accommodations should be requested on an individual basis, for example, some persons would need ramps while others would need Braille.

The third theme concerned accommodations that focus on facilitating communication. Here, most of the participants’ suggestions related to making available ‘human resources’. These included a ‘communication translator’ (this person was described as somebody who could interpret the person’s communication attempts if the person had no means of verbal communication and not in the more commonly understood form of a language translator); intermediaries; interpreters where necessary (i.e. persons translating one language into another, which could also include sign language interpreters) and allowing a ‘close person’ to be present for support. These ‘close persons’ were described as caregivers, close friends, guardians or family members.

Under the category of questioning, the participants described several different accommodations such as avoiding leading questions, giving questions in written format, making use of closed questions that would only require a yes/no response and using easy-to-read materials. They also discussed how the cross-examination process could be adapted (e.g. by asking the court to be more lenient with victims with communication disabilities; to assist the victim during cross-examination; for the prosecutor to provide the questions to the judge in written format so that the judge could ask them in a way that would not traumatise the person). Participants further stated that perhaps the questions (as well as the answers) could be presented in different formats (e.g. in electronic format) although they were not sure exactly what these alternative formats could be.

The fifth theme related to making provision for remote testimony, including video testimony from a different location and conducting proceedings at a different location, such as the witness’s home. (The reader is reminded that this NGT was conducted in the aftermath of the global coronavirus disease 2019 [COVID-19] pandemic.) The sixth and final theme addressed other accommodations that could be provided either before or after the court proceedings, including providing counselling before and after the court appearance, or where appropriate, alternative placement to prisons in the case of accused persons with disabilities who have been convicted.

Hereafter, the themes were further reduced, regrouped and renamed by the participants. After intensive discussion, the participants agreed on four main themes:

- Human accommodations to facilitate communication (e.g. intermediaries, interpreters, support persons such as a family member and translators)
- Environmental accommodations (e.g. closed court, in-camera proceedings in the judge’s chamber, ramp access, signage and restricted media access)
- Procedural accommodations (e.g. relaxed questioning strategies, written statements)
- Accommodations related to processes before (e.g. how bail hearings are conducted) and after the court appearance (e.g. sentencing and counselling).

## Discussion

The NGT discussion confirmed the findings from the biographic questionnaire as it became clear that the participants had not received any lectures about the provision of accommodations to guarantee equitable access to justice for individuals with communication disabilities. They expressed disappointment at not having received any lectures on the topic of accommodations and expressed a desire to learn more about disability. This is in line with a Canadian study that found that law students were generally very receptive to learning about disability and that professors expressed a keen interest in broadening their understanding in this field and exposing students to it (Lepofsky 2022).

Nevertheless, the number of Post-it notes generated during the NGT indicated the richness of the participants’ voices. Despite not having had specific lectures on disability, final-year students were able to draw on knowledge gleaned during their studies and provide a comprehensive range of different accommodations. As the students were familiar with one another, rapport was quickly established, thus creating a non-threatening environment, which yielded rich and diverse data. The students were eager to explain and justify their ideas, and being surprised that they were more aware of accommodations than they had initially anticipated, they suggested a comprehensive range of accommodations. Furthermore, although none of the participants had yet been to court, this did not negatively impact their knowledge of accommodations.

Although the participants did not know much about disability, they had some knowledge about accommodations. They based this knowledge on having a family member with a disability or having watched popular television shows in which persons with disabilities are featured (such as The Good Doctor, which stars an autistic medical doctor). To conceptualise various accommodations, the participants also drew parallels with accommodations for children. Furthermore, they asked questions about the various accommodations to relate this to existing knowledge, indicating that they were internalising the information (Lepofsky 2022). They related the accommodations to the rules of criminal procedure and evidence taught in procedural law, which is commonly recognised as a fundamental element in the study of law (Du Plessis & Welgemoed 2022). This led them to asking pertinent questions such as whether some of the accommodations they suggested, such as providing written testimony instead of oral testimony, would be permitted in court.

The nominal group discussion also revealed that the participants’ knowledge was compartmentalised in terms of the modules they had studied. For instance, when in camera proceedings were mentioned, they immediately related this to their civil procedure module where the Socratic pedagogy is applied. It is typically used during the training of undergraduate law students where, according to Du Plessis and Welgemoed (2022), they compartmentalise their work into ‘neat and artificial categories’. Crucially, the participants did not object to the provision of any of the accommodations, for example, on the basis of reasonableness. Instead, they were accepting of the fact that accommodations need to be considered on a case-by-case basis, which is in line with best practices in disability (White et al. 2020). This finding highlights the fact that at an undergraduate level, students need to be taught how to view a law topic through a disability lens (Lepofsky 2022). For instance, when addressing the Bill of Rights, students ought to learn that, for individuals with communication disabilities, the right to equality before the law includes acknowledging the right to access justice, particularly through the provision of various accommodations.

Universities should be encouraged to design an LLB curriculum that provides the most comprehensive and professional preparation for law students in anticipation of legal practice. Consistent with Article 9 of the CRPD, stakeholders should undergo training to address accessibility issues affecting individuals with disabilities (United Nations 2006).

Not all law students will opt for a career in litigation after obtaining their LLB degree. Nonetheless, the fact that they have legal training may lead many to professions involving litigation, such as serving as legal advisers or corporate lawyers. In these roles, they may act as the primary interface between their employers and practising attorneys or advocates. The chances that they will encounter persons with communication disabilities are significant. The primary responsibility of universities should be to determine the essential knowledge that lawyers, as members of a privileged profession, must possess at a minimum. This necessitates knowledge about disability. Students should at least be equipped with basic disability literacy skills, which refers to the fact that they should be educated about the components of disability etiquette, encompassing appropriate ways to address and engage with individuals who have disabilities (Van Niekerk, Maguvhe & Magano 2022). There should be an awareness of the need for physical accessibility (e.g. ramps at court buildings, chambers and bathrooms), as well as the techniques that guarantee effective communication with individuals with disabilities, both in face-to-face interactions and over the phone. Law students should be made aware of the procedural accommodations that could be made available to support persons with communication disabilities (White & Msipa 2018). This implies adequate internal and organisational capacity to provide accommodations when requested or needed. While disability literacy should be seen as a core skill for LLB students, a disability curriculum should be provided to all law students, not exclusively to those who are enrolled in specialised disability courses (Lepofsky 2022). Within the legal context, disability literacy entails the ability to acknowledge the necessity for and pinpoint the distinct services and benefits that individuals with disabilities might need. It also involves developing the capability to assist them in evaluating and understanding their options for accessing justice.

## Limitations

Small sample sizes are often critiqued for limited generalisability of the results, but using an NGT focusses on minimising the ‘participation paradox’ (that is where an increase in participants diminishes the role of the individual) by intentionally choosing a small sample and leveraging the strengths of both individual and group interviews (Giuliani et al. 2023; Naudé & Bornman 2021). To contain minimal group studies and ensure an effective yet manageable process, the group was restricted to a maximum of seven participants (McMillan et al. 2014). It should also be noted that the participants had strong personal experiences of disability in their own lives; hence they may have been more informed and thoughtful on the topic than their peers.

## Conclusions

Students in their final year of undergraduate studies have a fundamental understanding of procedural law as the latter forms the basis of their law training in the years that preceded. Expanding on that, they could be guided to understand and appreciate disability rights to know how to apply the procedural law principles to serve as a vehicle for understanding how accommodations can be provided in line with human rights law. Making disability inclusion a focal point in the education of law students is essential. However, during undergraduate training, there is often little to no practical application as to how the laws and principles would manifest in a courtroom.

The Socratic method, which has been described as the signature training pedagogy for law students globally, focusses on classroom lectures with an emphasis on students solely engaging with theoretical concepts. At the core of these lectures lies discussion, complemented by the utilisation of textbooks, legislation and occasionally, authentic legal documents. Questions, answers and discussions revolve around the topics addressed at a particular stage of the course (Du Plessis & Welgemoed 2022). The Socratic method additionally necessitates lecturers to craft and organise questions, along with their responses to students’ questions, intending to steer students towards solutions. This presupposes that such an approach fosters critical thinking and the proficient presentation of ideas and responses to the relevant questions. However, this approach incorporates minimal or no practical application to simulated or real-life situations (Lepofsky 2022). Hence, students enter legal practice without experience of how to consider – from a holistic perspective – all evidence in a particular case (e.g. from a witness with a communication disability).

Elevating disability rights to have a consistent, robust and equitable emphasis in undergraduate law education is critical, aligning with the principles of equity, diversity and inclusion emphasised in the Constitution. It is critical that all personnel involved in the administration of justice, including police officers, lawyers, prosecutors, magistrates and judges, possess a certain understanding of how to promote and facilitate the effective participation of individuals with communication disabilities through the provision of accommodations throughout the legal process. Ideally, the impartation of this knowledge should start at the undergraduate level when pre-service legal practitioners are receiving legal practice training (Du Plessis & Welgemoed 2022).

At present, the legal professionals in South Africa lack adequate preparation to address the legal requirements of individuals with communication disabilities. This gap arises because legal education has predominantly, if not exclusively, concentrated on training undergraduate law students to cater to individuals without disabilities. In fact, law students can complete their legal studies without gaining any knowledge on how to fulfil the legal requirements of clients with disabilities by providing accommodations (Lepofsky 2022). Typically, disability rights and access to justice for persons with disabilities (and by implication communication disability) are taught at the Master’s level at South African universities, and most practising lawyers in South Africa do not hold a Master’s degree. Consequently, newly graduated lawyers entering the workforce possess minimal to no knowledge about offering accommodations to individuals with communication disabilities within the justice system.

The plea in this article is therefore that law students should be exposed to disability challenges as part of their training, for example, through conducting tutorial sessions on disability, verbal arguments, mock trials and the presentation of evidence. Mock trials can be in the format of brief class exercises or allocated dedicated time, for instance playing out a particular scenario about a person with a disability.

In summary, the research objectives and discussed themes led to the following conclusions:

- The education on disability law at the university level should be designed to mould students into competent and professionally-minded legal practitioners capable of delivering high-quality legal services to all citizens, including persons with (communication) disability (Du Plessis & Welgemoed 2022). This involves a solid understanding of the accommodations that can be offered to ensure substantive justice for these individuals.
- In recent times, the prominence of disability rights and the significance of ensuring access to justice for all have increased, particularly with the adoption of the CRPD (United Nations 2006), and in particular its Article 13, which highlights the impact of international legislation.

## Recommendations

The following recommendations emerged from the findings of the NGT:

- Disability rights must be included in the undergraduate LLB curriculum because law students often graduate without fundamental practical skills in disability law, hindering their ability to ensure access to justice for all in their professional practice.
- There is a need to raise awareness about the importance of court accommodations, not only among undergraduate law students but also among those who are already in legal practice and persons from other sectors, including academia, organisations of persons with disabilities and law and policymakers.
- Domestic legislation, which may not consistently offer accommodations in court for individuals with communication disabilities, should be reviewed and amended to ensure that it expressly mandates the provision of accommodations.
- In some jurisdictions, accommodations are included in practice bench books. It would be suitable to also include these in the South African context.
